# CD33 expression combined with D15-MRD positivity identifies poor prognosis in children with *ETV6*::*RUNX1*-positive ALL

**DOI:** 10.1007/s00277-026-07089-8

**Published:** 2026-05-29

**Authors:** Xueling Zheng, Yuxi Luo, Ruidong Zhang, Xiaohang Liu, Pengli Huang, Chao Gao, Hui Chen, Jia Fan, Wei Lin, Jiaole Yu, Yuanyuan Zhang, Peijing Qi, Ying Wu, Xiaoxi Zhao, Jun Li, Xiaoxia Peng, Tianyou Wang, Huyong Zheng

**Affiliations:** 1 Hematology Center, National Key Discipline of Pediatric Hematology, Key Laboratory of Major Diseases in Children, Beijing Children’s Hospital, National Key Discipline of Pediatrics (Capital Medical University), Ministry of Education, Capital Medical University, National Center for Children’s Health, Beijing, 100045 China; 2 Center for Clinical Epidemiology and Evidence-based Medicine, Beijing Children’s Hospital, Capital Medical University, National Center for Children’s Health, Beijing, 100045 China; 3 Department of Clinical Laboratory Center, National Key Clinical Discipline of Pediatric Hematology, National Key Discipline of Pediatrics (Capital Medical University), Key Laboratory of Major Diseases in Children, Ministry of Education, Beijing Children’s Hospital, Capital Medical University, National Center for Children’s Health, Beijing, 100045 China

**Keywords:** Pediatric, Acute lymphoblastic leukemia, *ETV6::RUNX1*, CD33, Prognosis

## Abstract

While *ETV6*::*RUNX1*-positive acute lymphoblastic leukemia (ALL) is generally associated with favorable outcomes, a subset of patients experience relapse despite receiving standard therapy, underscoring the need for early biomarkers to identify high-risk subgroups. In this retrospective study of 345 consecutive pediatric patients with *ETV6*::*RUNX1*-positive ALL treated in accordance with the protocol of the Chinese Children’s Leukemia Group (CCLG-ALL 2008), CD33 expression (CD33^+^) on leukemic blasts was detected in 55.9% of patients and was significantly associated with minimal residual disease (MRD) positivity on day 15 (D15-MRD-positive) (65.2% vs. 42.0%, *P* < 0.001). Multivariate analysis revealed CD33^+^ status as an independent risk factor for D15-MRD positivity (odds ratio (OR) = 2.66, 95% confidence interval (CI) 1.66–4.26). Among CD33^+^ patients, those who were D15-MRD-positive had significantly inferior 5-year and 10-year event-free survival (EFS) relative to those who were D15-MRD-negative (5-year EFS: 91.9% ± 2.4% vs. 100%, *P* = 0.027; 10-year EFS: 89.2% ± 3.0% vs. 100%, *P* = 0.014). This prognostic association was not observed in CD33-negative patients. In conclusion, the combination of CD33^+^ and D15-MRD-positivity may identify a distinct high-risk subgroup within *ETV6*::*RUNX1*-positive ALL. Early intervention, potentially including CD33-directed therapy, may represent a promising strategy to improve outcomes in this subgroup, although further validation is warranted.

**Supplementary Information:**

The online version contains supplementary material available at 10.1007/s00277-026-07089-8.

## Introduction

*ETV6*::*RUNX1*-positive ALL accounts for approximately 25% of pediatric B-ALL cases. Although the “two-hit” theory partially explains the pathogenesis of *ETV6*::*RUNX1*-positive ALL and also reveals its clinical and biological heterogeneity to a certain extent [1–3], the correlation between such heterogeneity and clinical prognosis has not been fully elucidated. Patients with distinct molecular subtypes, unfavorable subclones, or early minimal residual disease (MRD) positivity show inferior treatment responses and higher relapse risks [4–6]. Despite an overall cure rate exceeding 90%, the absolute number of relapses per year is still high because of the large proportion of this group among the total ALL patients. Moreover, late relapse (3–10 years after treatment cessation) poses challenges for prognostic research [7, 8]. Given optimistic clinical expectations, relapse after long-term remission is particularly devastating. Therefore, in the context of these clinical challenges, it is highly important to explore the prognosis-related heterogeneity of *ETV6::RUNX1*-positive ALL and to provide evidence for targeted early intervention strategies. In this study, data from 345 children with *ETV6::RUNX1*-positive ALL admitted to our hospital between August 2008 and August 2018 were analyzed retrospectively, with a focus on the relationships between the heterogeneity of clinical characteristics at initial diagnosis and both early chemotherapy response and long-term prognosis.

## Methods

### Patient characteristics

This was a single-center retrospective cohort study. Children with *ETV6::RUNX1*-positive ALL who were initially treated at Beijing Children’s Hospital, Capital Medical University, National Center for Children’s Health between August 2008 and August 2018 were enrolled. Overall ALL diagnosis was established on the basis of bone marrow morphology, immunology, cytogenetics, and molecular (MICM) criteria. The *ETV6::RUNX1* fusion subtype was confirmed by molecular testing (real-time quantitative PCR) and/or FISH analysis. Given the targeted research purpose, patients without the *ETV6::RUNX1* fusion gene were excluded from the present cohort and not analyzed in this study.

The inclusion criteria were as follows: (1) onset before18 years of age; (2) newly diagnosed, previously untreated ALL confirmed by MICM classification; (3) positive *ETV6::RUNX1* fusion verified by molecular biology or FISH; and (4) standardized risk assessment, chemotherapy and regular follow-up carried out in accordance with the CCLG-ALL 2008 regimen. The exclusion criteria were as follows: (1) clinical data at the time of initial diagnosis could not be obtained; (2) comorbidities (other tumors or immune diseases); and (3) received chemotherapy or targeted drugs other than those recommended in the CCLG-ALL 2008 protocol (see next section).After applying the unified inclusion criteria, a total of 361 *ETV6::RUNX1*-positive patients were initially enrolled in this study. 16 patients were excluded after applying the exclusion criteria. Ultimately, 345 patients were included as research subjects. In the regimen, patients aged ≥ 10 years are defined as adolescents.

Written informed consent was obtained from the parents or guardians of the children who served as the study subjects. This study was conducted in accordance with the Declaration of Helsinki and approved by the Institutional Review Board (IRB) of Beijing Children’s Hospital, Capital Medical University, National Center for Children’s Health.

### Treatment regimen and follow-up

All patients in this cohort harbored the *ETV6::RUNX1* fusion gene, a favorable genetic aberration conventionally classified as a standard risk (SR) factor. The risk stratification in this study was comprehensively determined in accordance with the CCLG-ALL 2008 protocol, which combines genetic features and clinical indicators; risk stratification using this protocol mainly relies on a two‑step staged evaluation system, including initial risk assessment and final risk reassessment. A total of 62 patients were initially classified as intermediate risk (IR) or high risk (HR) because of clinical high-risk factors, including age ≥ 10 years, initial white blood cell count ≥ 50 × 10⁹/L, and poor prednisone response; thus, these patients received risk-adapted corresponding chemotherapy. All remaining patients were initially assigned to the SR group. Bone marrow remission status was defined as M1 (immature cells in bone marrow < 5%), M2 (5% ≤ immature cells in bone marrow < 25%), or M3 (immature cells in bone marrow ≥ 25%). The induction protocol for the SR group included VDLD_2_ (daunorubicin on day 8 and day 15), vincristine (1.5 mg/m^2^/d), dexamethasone (6 mg/m^2^/d), L-asparaginase (5000 U/m^2^/d) and daunorubicin (30 mg/m^2^/d); the induction protocol for the IR and HR groups included VDLD_4_ (daunorubicin on days 8, 15, 22, and 29), vincristine, dexamethasone, L-asparaginase and daunorubicin (same dose as the SR group). Please refer to a previous study for the overall scheme [9].

Since the three induction schemes were exactly the same before day 15 and the induction schemes for IR and HR groups were the same before day 33 (two more doses of daunorubicin were provided on day 22 and day 29; these doses were not provided in the SR group), the following indicators at initial diagnosis and within 15 days were analyzed as a whole group. Patients with or without indicators measured after 15 days but within 33 days were divided into the IR/HR group and SR group, respectively, for data analysis. Surviving patients were followed up until July 1, 2025, and follow-up was terminated when patients died. Subsequent updated protocols were exclusively used for salvage therapy in patients who relapsed after August 2018. The 2018 salvage protocol retained the same core chemotherapy (VDLP, CAM, high‑dose MTX/cytarabine) recommendation as that recommended in the CCLG-ALL 2008 protocol, with only minor adjustments: L‑asparaginase was optimized to PEG‑asparaginase, optional rituximab was added (nonmandatory), and supportive care, anti-infection treatment and HSCT suitability were optimized; all frontline induction and consolidation remained strictly in accordance with the CCLG-ALL 2008 protocol.

### Main equipment and reagents for laboratory investigations

① Flow cytometry for the immunophenotyping of blasts: A BD FACSCanto II flow cytometer (Becton Dickinson, USA) was used. The following monoclonal antibodies (all purchased from BD Pharmingen or Beckman Coulter (USA)) were used: anti-CD5, anti-CD7, anti-CD41, anti-CD2, anti-Kappa, anti-Lambda, anti-MPO, anti-cyIgM, anti-cyCD3, anti-CD10, anti-HLA-DR, anti-cyCD79a, anti-CD13, anti-CD22, anti-CD33, anti-CD34, anti-CD19, anti-CD38, anti-CD123, anti-CD20, anti-CD117 and anti-CD56. The criteria for positivity included an expression rate ≥ 20% for lymphoid, myeloid and nonlineage-specific antigens and an expression rate ≥ 10% for cyCD3, cyCD79a and MPO.
② Flow MRD detection: Four-color flow cytometry (BD FACSCanto II) was performed for B-lineage ALL MRD monitoring, following the classical 4-color panel (widely validated and recommended by early EuroMRD consensus, 2000). Antibody panels were purchased from BD Pharmingen or Beckman Coulter (USA).
③ Quantitative real-time polymerase chain reaction (PCR) (qRT‒PCR) for molecular MRD detection: Molecular MRD detection was performed on an ABI ViiA 7 Real-Time PCR System (Applied Biosystems, USA) in strict accordance with EuroMRD Consortium Guidelines for ETV6::RUNX1-positive childhood ALL (Supplementary Data 1).
④ Criteria for MRD positivity: D15-MRD > 0.1% was defined as D15-MRD‑positive, and D33-MRD > 0.01% was defined as D33-MRD‑positive.

### Statistical analysis

IBM SPSS Statistics 23.0 statistical software and R 4.1.2 were used for statistical analysis. Normally distributed data are presented as the mean ± standard deviation, whereas nonnormally distributed data are presented as the median (upper and lower quartile). For continuous variables, the data were analyzed using the *t* test and Mann‒Whitney *U* test, depending on the data distribution. Count data are presented as the number of cases or percentages. For categorical variables, the data were analyzed using the chi-square test. Event-free survival (EFS) was estimated from the date of diagnosis to the date of relapse, death or last contact with the patient. Overall survival (OS) was defined as the time from the date of diagnosis through the date of death for any reason. The Kaplan‒Meier method and the log-rank test were used for the survival analysis. Logistic regression was used for multivariate analysis. The ability of CD33⁺/D15-MRD⁺ to predict D33-MRD outcomes was evaluated via receiver operating characteristic (ROC) curve analysis among patients in the SR group. P values < 0.05 were considered to indicate statistical significance. A Gini index decision tree was used to explore important prediction factors for the prognosis of *ETV6::RUNX1*-positive patients based on CD33 test results (positive or negative), and random oversampling was used to generate new balanced data for better effectiveness. To avoid overfitting of the decision tree, the mini-split was controlled to be more than 20 and 10 in the CD33-positive and -negative subgroups, respectively.

## Results

### General characteristics of the patients (Fig. 1)

A total of 345 children with *ETV6::RUNX1*-positive ALL were included in this study. The ratio of SR to IR to HR patients in the preliminary assessment was 141.5:30.0:1.0, corresponding to 82.0%, 17.4%, and 0.6% of the total patients, respectively, and the ratio of SR to IR to HR patients in the final assessment was 12.1:11.5:1.0, corresponding to 49.2%, 46.7%, and 4.1% of the total patients, respectively. In terms of prognosis, 19 patients experienced adverse clinical events during follow-up. 16 patients developed leukemic relapse as the primary event, among whom 2 eventually died. The remaining 3 events were disease progression-related deaths without prior relapse. Among the 16 patients with disease relapse, 7 experienced relapse after August 2018 and received salvage chemotherapy with the updated protocol, with one death occurring after salvage treatment. Notably, all 5 deaths were exclusively disease-related, and no treatment-related toxicity deaths or infection-related deaths occurred in this cohort. A total of 15 adolescents were enrolled in this study, among whom only one experienced an event. The overall EFS rates at 5 years and 10 years were 95.0% ± 1.2% and 93.9% ± 1.4%, respectively. Deaths occurred within 5 years, and the overall survival (OS) rate was 98.6% ± 0.6%.

**Fig. 1:**
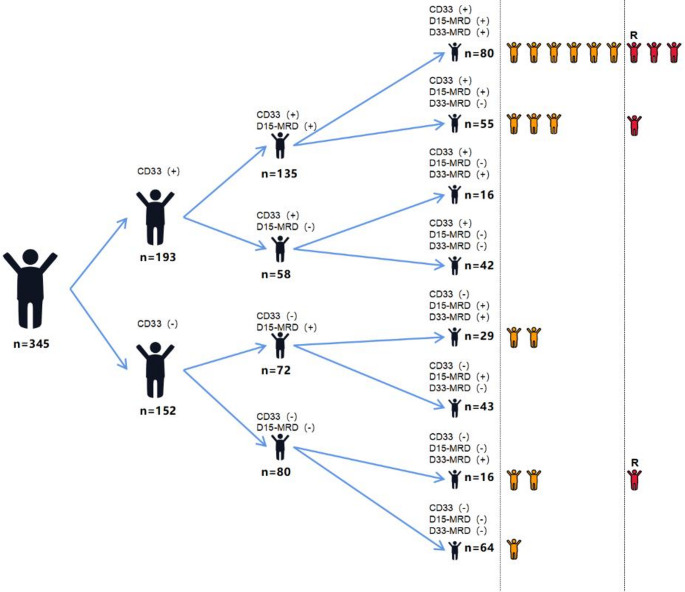
Adverse Events in Children. Below the human-shaped icons, “n” indicates the number of individuals in each group; group labels are above the icons. Orange icons represent children with relapsed adverse events, where the icon count reflects the number of such occurrences in their respective groups. Red icons denote children experiencing fatal adverse events, with the icon count indicating the total number of deaths in each group. The “R” above the red icons signifies that the child died after relapse

### Analysis of MRD and prognosis in different risk groups

In this study, the incidence of clinical events in the D15-MRD-positive group tended to be greater than that in the D15-MRD-negative group (7.2% vs. 2.9%, *P* = 0.083). Both the 5-year (93.7% ± 1.7% vs. 97.0% ± 1.5%, *P* = 0.156) and 10-year (91.9% ± 2.1% vs. 97.0% ± 1.5%, *P* = 0.084) EFS rates in the positive group were lower than those in the negative group, but the difference was not statistically significant. After the risk assessment on day 15, a total of 283 children were initially assessed as SR, one of whom died before the 33rd day; thus, there was no D33-MRD result for that patient. The incidence of clinical events in the D33-MRD-positive group was greater than that in the D33-MRD-negative group (9.9% vs. 1.8%, *P* = 0.002), and the 5-year (91.8% ± 2.6% vs. 98.2% ± 1.0%, *P* = 0.010) and 10-year (89.0% ± 3.2% vs. 98.2% ± 1.0%, *P* = 0.003) EFS rates were both lower in the D33-MRD-positive group than in the D33-MRD-negative group. In the IR/HR group (*n* = 62), all clinical events occurred within 3 years after diagnosis. There was no significant difference in the 5-year EFS or 10-year EFS (93.1% ± 4.7% vs. 93.9% ± 4.2%, *P* = 0.908) between the D33-MRD-positive and D33-MRD-negative groups.

### Correlation between immunophenotypic heterogeneity and MRD

In this study, the above results revealed a correlation between MRD and prognosis. *ETV6::RUNX1*-positive ALL is a type of leukemia with significant intragroup heterogeneity. In clinical work, we observed that different immunophenotypes may be associated with MRD (Table 1; Supplementary Data 2).

**Table 1:** Univariate variable analysis of D15-MRD and D33-MRD

Immunotype	Total (*n* = 345)					SR (*n* = 282)^a^					IR + HR (*n* = 62)				
	*N*	%	MRD-D15 positive	MRD-D15 negative	*P*	*N*	%	MRD-D33 positive	MRD-D33 negative	*P*	*N*	%	MRD-D33 positive	MRD-D33 negative	*P*
Myeloid marker ^b^	209	60.6%	69.6%	47.1%	<0.001	176	62.4%	73.9%	55%	0.001	32	51.6%	58.6%	45.5%	0.301
CD10	343	99.4%	99.5%	99.3%	>0.999	280	99.3%	99.1%	99.4%	>0.999	62	100%	-	-	-
CD13	17	4.9%	6.8%	2.2%	0.054	16	5.7%	7.2%	4.7%	0.370	1	1.6%	0%	3%	>0.999
CD20	49	14.2%	14.0%	14.5%	0.900	40	14.2%	10.8%	16.4%	0.191	9	14.5%	10.3%	18.2%	0.483
CD22	129	37.4%	37.2%	37.7%	>0.999	109	38.7%	28.8%	45.0%	0.006	20	32.3%	17.2%	45.5%	0.018
CD33	193	55.9%	65.2%	42.0%	<0.001	162	57.4%	70.3%	49.1%	<0.001	30	48.4%	58.6%	39.4%	0.131
CD34	334	96.8%	97.1%	96.4%	0.950	275	97.5%	97.3%	97.7%	>0.999	58	93.5%	86.2%	100%	0.043
CD38	1	0.3%	0%	0.7%	0.400	1	0.4%	0%	0.6%	>0.999	0	0%	-	-	-
CD56	57	16.5%	14.0%	20.3%	0.124	49	17.4%	9.9%	22.2%	0.008	8	12.9%	6.9%	18.2%	0.264
CD117	37	10.7%	10.1%	11.6%	0.670	32	11.3%	9.0%	12.9%	0.319	5	8.1%	6.9%	9.1%	>0.999
cyCD79a	343	99.4%	100%	98.6%	0.159	280	99.3%	99.1%	99.4%	>0.999	62	100%	-	-	-
cyIgM	14	4.1%	1.9%	7.2%	0.014	11	3.9%	3.6%	4.1%	>0.999	3	4.8%	6.9%	3.0%	0.595

^a One patient died before D33 assessment, so there was no D33 bone marrow MRD result; ^b. Myeloid markers are CD13 or CD33 positive

**Fig. 2:**
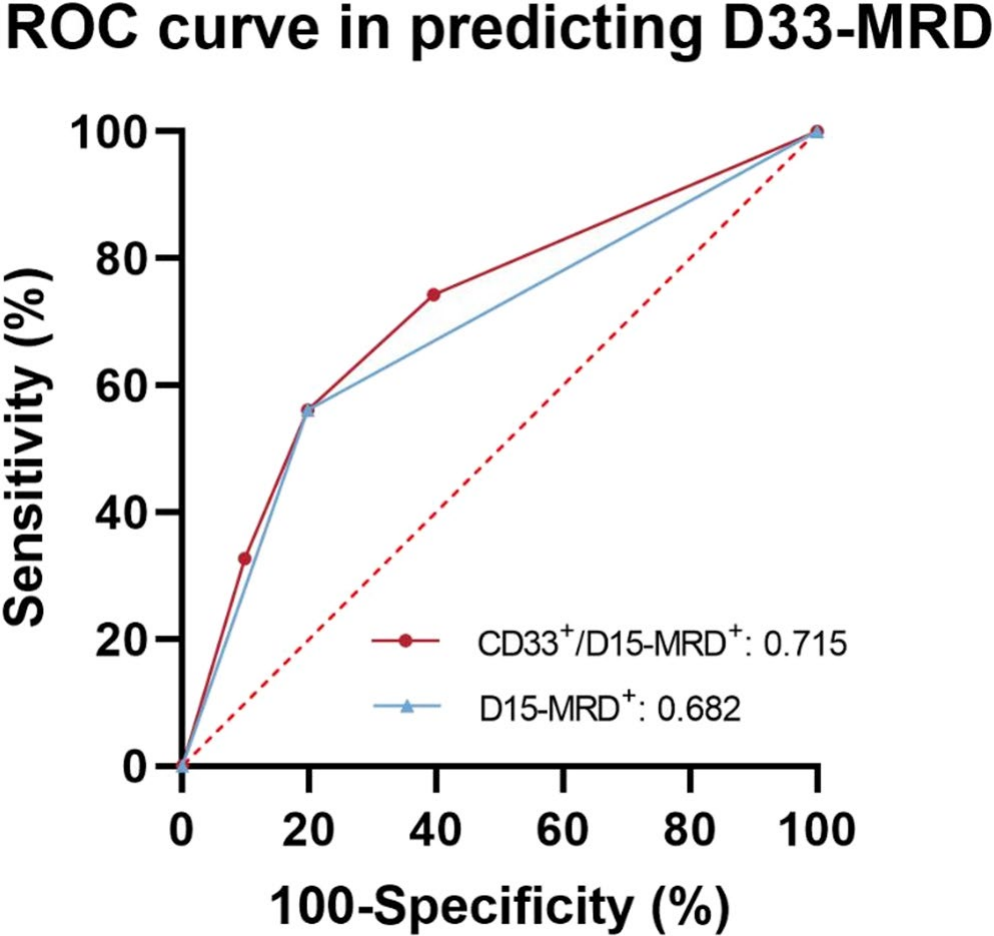
Comparison of ROC curves for CD33^+^/D15-MRD^+^ and D15-MRD^+^ for predicting D33-MRD outcomes

All patients had CD19 and HLA-DR immune markers in blasts; 60.6% of patients had CD13-positive (CD13^+^) and/or CD33-positive (CD33^+^) myeloid-labeled blasts, and CD33^+^ blasts were present in 55.9% of patients. Patients with CD33^+^ blasts were more common in the D15-MRD-positive group than in the D15-MRD-negative group (65.2% vs. 42.0%, *P* < 0.001). Among the SR patients, the incidence of patients with CD33^+^ blasts was greater in the D33-MRD-positive group than in the D33-MRD-negative group (70.3% vs. 49.1%, *P* < 0.001). Furthermore, for predicting D33-MRD positivity, the combination of CD33 expression and D15-MRD positivity (CD33^+^/D15-MRD^+^) appears to yield better predictive performance than D15-MRD positivity alone (Fig. 2). CD22-positive (CD22^+^) blasts were present in 37.4% of the patients. In the SR group and IR/HR group, the incidence of patients with CD22^+^ blasts was lower in the D33-MRD-positive group than in the D33-MRD-negative group (28.8% vs. 45.0%, *P* = 0.006; 17.2% vs. 45.5%, *P* = 0.018).

All the univariate variables with significant differences were analyzed by logistic regression. CD33^+^ blasts in the bone marrow [odds ratio (OR) (95% confidence interval (CI)) = 2.658 (1.660–4.256)] was an independent risk factor for D15-MRD positivity. In the SR group, CD33^+^ blasts in the bone marrow was an independent risk factor for persistent D33-MRD positivity [OR (95% CI) = 2.240 (1.336–3.757)].

### Analysis of the CD33-stratified decision tree

**Fig. 3:**
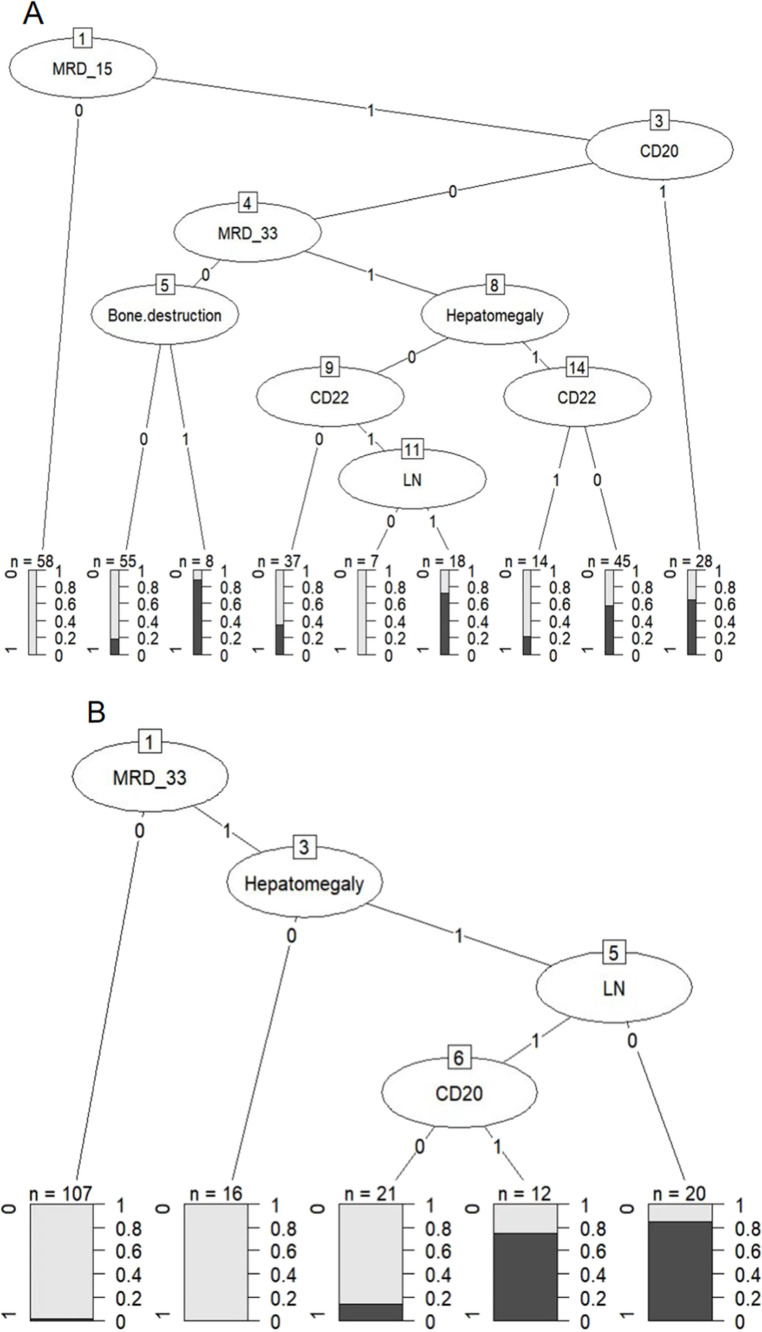
Decision Tree Prediction Model. MRD_15 represents the MRD status on day 15 (1 = positive, 0 = negative), and MRD_33 represents the MRD status on day 33 (1 = positive, 0 = negative). LN indicates lymph node status (including superficial and deep lymph nodes), with 1 defined as lymphadenopathy and 0 as normal. (**A**) Long-term prognosis decision tree model for CD33-positive patients; (**B**) long-term prognosis decision tree model for CD33-negative patients

On the basis of the above results, stratified by CD33 expression status, 10 of the 16 patients with disease relapse were CD33 positive, while the remaining 6 were CD33 negative. Among the five disease-related deaths, four occurred in the CD33-positive subgroup, and only one death occurred in the CD33-negative subgroup. This unbalanced distribution indicates that CD33 status should be taken into full consideration during initial risk stratification at diagnosis. Furthermore, CD33 may serve as a valuable auxiliary index for subsequent dynamic risk re-evaluation throughout treatment. Therefore, we conducted a prognosis decision tree analysis (Fig. 3). The decision tree revealed that D15-MRD and D33-MRD were first-order predictors in CD33^+^ and CD33^−^ patients (Supplementary Data 3).

### Relationship between CD33 expression and prognosis

In all patients with CD33^+^ blasts (*n* = 193), both the 5-year (91.9% ± 2.4% vs. 100%, *P* = 0.027) and 10-year (89.2% ± 3.0% vs. 100%, *P* = 0.014) EFS rates were lower in the D15-MRD-positive group than in the negative group. However, among patients without CD33^+^ blasts (CD33^−^), there was no significant difference in EFS rates between the D15-MRD-positive and D15-MRD-negative groups (97.2% ± 2.0% vs. 94.9% ± 2.5% at 5 and 10 years, *P* = 0.464). There was no effect of D33-MRD on 10-year EFS in patients with CD33^+^ blasts (SR: *P* = 0.085; IR/HR: *P* = 0.373).

**Fig. 4:**
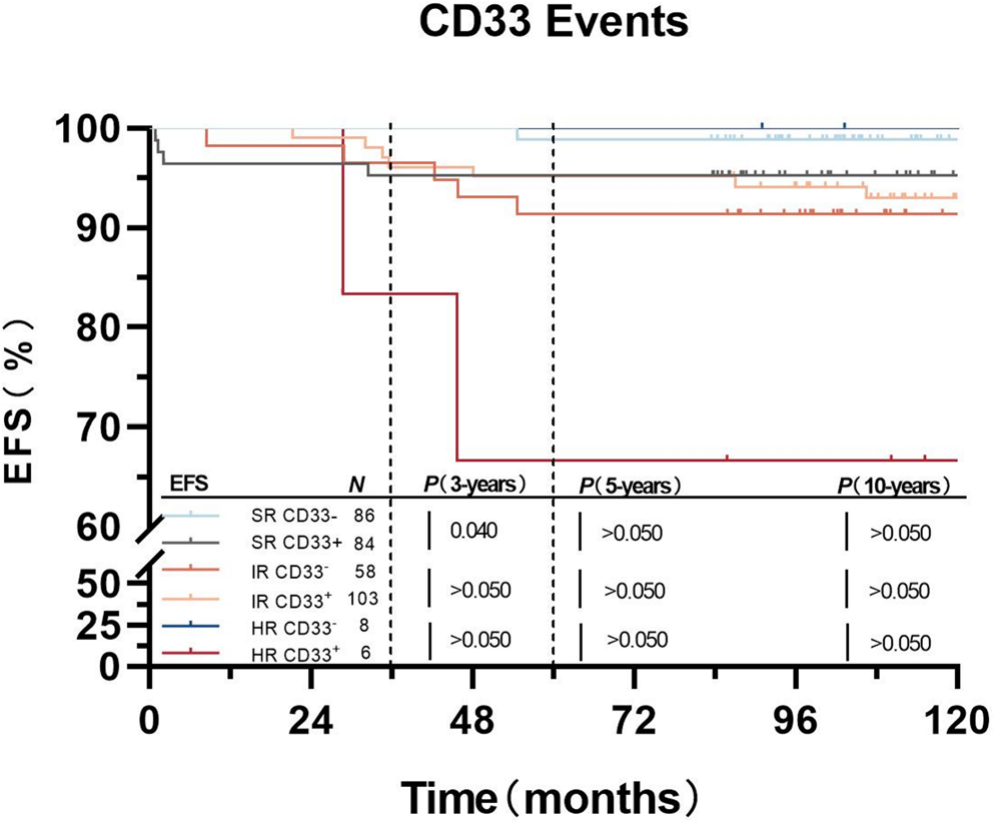
EFS of *ETV6*::*RUNX1*-positive ALL patients with different CD33 expression levels in different risk groups

To explore the relationship between CD33 expression and long-term prognosis, we conducted a hierarchical comparison according to different chemotherapies for different clinical risks. For those who received SR chemotherapy, the 3-year EFS was lower in the CD33^+^ group than in the CD33^−^ group (95.2% ± 2.3% vs. 100%, *P* = 0.041), but there was no significant difference in the 5-year or 10-year EFS (95.2% ± 2.3% vs. 98.8% ± 1.2%, *P* = 0.161). For those who received IR chemotherapy, the 10-year EFS was not significantly different between the CD33^+^ group and the CD33^−^ group (92.2% ± 2.9% vs. 91.3% ± 3.7%, *P* = 0.613). For those who received HR chemotherapy, the 10-year EFS tended to be greater in the CD33^+^ group than in the CD33^−^ group (66.7% ± 19.2% vs. 100%, *P* = 0.087) (Fig. 4).

**Fig. 5:**
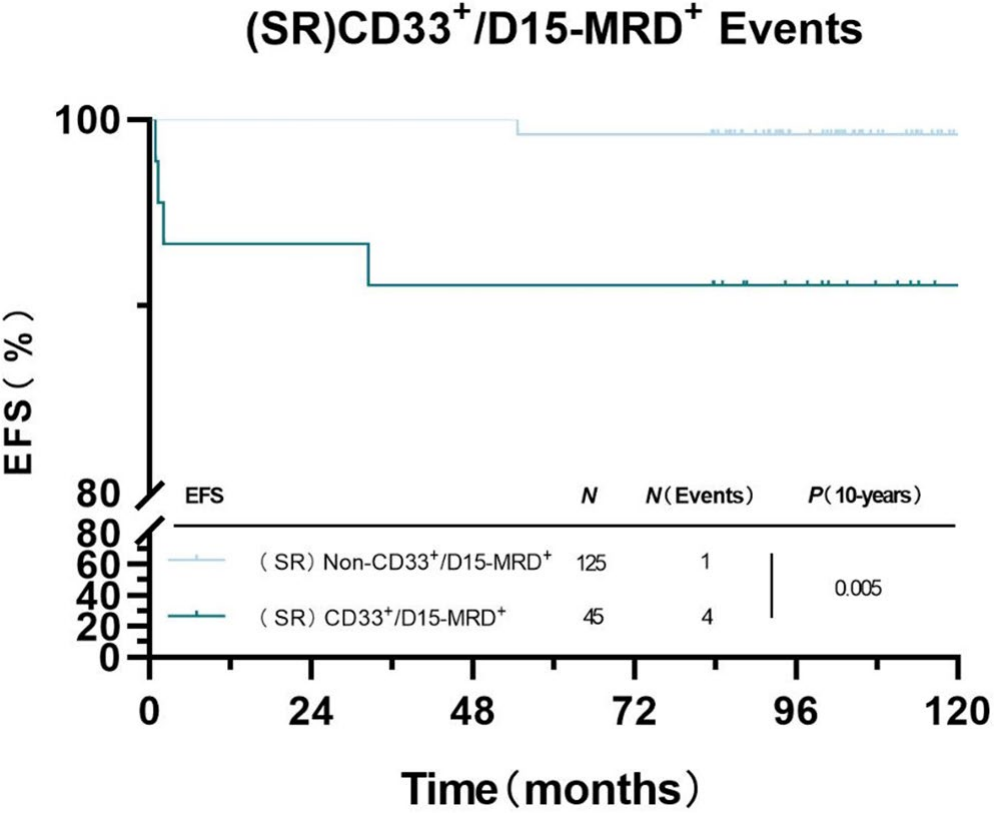
EFS of *ETV6*::*RUNX1*-positive ALL patients according to CD33^+^/D15-MRD^+^ status in the standard-risk group

**Fig. 6:**
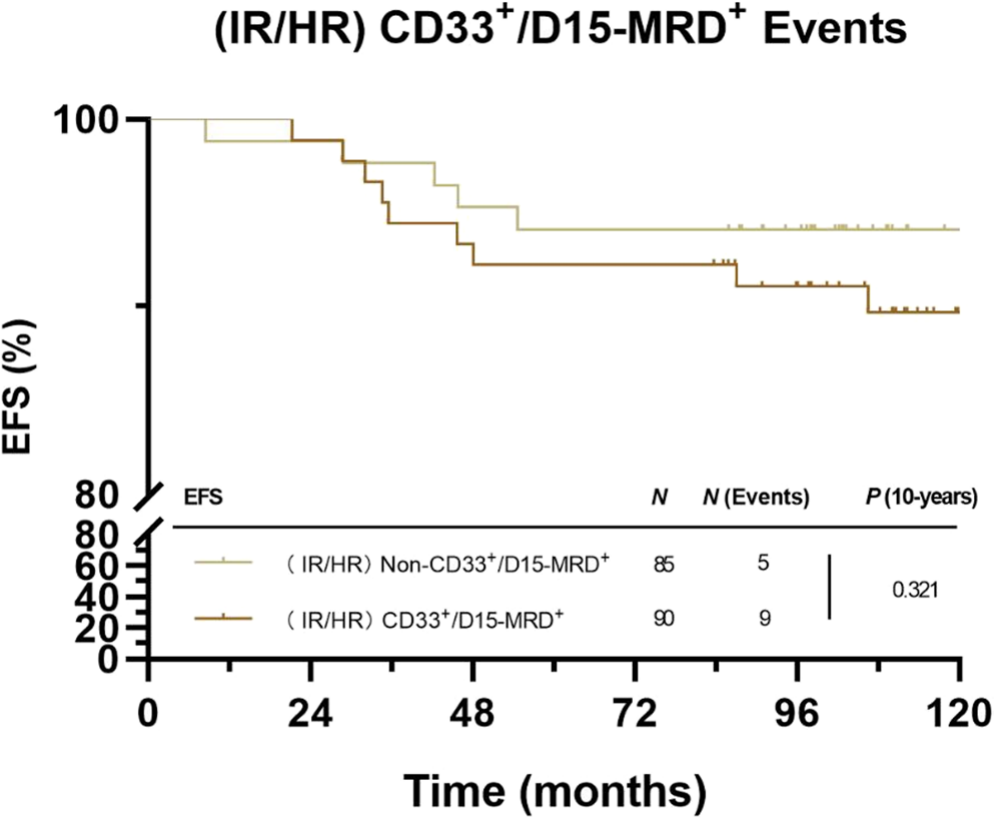
EFS of *ETV6*::*RUNX1*-positive ALL patients according to CD33^+^/D15-MRD^+^ status in the intermediate-risk group or high-risk group

Our findings indicate that CD33 has limited utility as a standalone predictive biomarker for risk stratification, and additional inclusion of CD22 does not significantly alter these results (Supplementary Data 4). On the basis of the decision tree analysis and the distribution of affected children, we observed that CD33^+^ patients within the SR group with MRD on day 15 (CD33^+^/D15-MRD-positive) exhibited significantly poorer event-free survival (Fig. 5). However, this association was not evident in either the IR or HR groups (Fig. 6). In the subgroup analysis, the HR CD33^+^ subgroup exhibited relatively inferior survival outcomes, i.e., 2 relapse events among 6 patients. Although the small event number may weaken the statistical power, this subtle prognostic difference still deserves clinical attention.

## Discussion

In pediatric *ETV6*::*RUNX1*-positive ALL, the clinical significance of heterogeneity at diagnosis—especially in terms of myeloid marker expression and early MRD status—with respect to early chemotherapy response and long-term prognosis remains incompletely understood. In this cohort study of 345 patients, we revealed that patients with CD33 expression on leukemic blasts at diagnosis and D15-MRD positivity may represent a high-risk subgroup for whom SR chemotherapy may not be sufficient. 

*ETV6*::*RUNX1*‑positive ALL is a well‑investigated subtype with a favorable prognosis. Our 5-year EFS and OS rates were 95.1% and 98.6%, respectively; our rates are comparable to those reported in a study conducted at St. Jude’s but slightly greater than those reported in several multicenter studies [6, 7, 10–12]. These differences may partly stem from more uniform treatment implementation, supportive care and complication management in single-center setting. Approximately 50% of patients achieved favorable outcomes with SR chemotherapy. However, 15 of 19 patients (78.9%) with clinical events were initially stratified as having an SR at diagnosis, and 5 remained in the SR group after reassessment. This finding indicates that pre‑day‑33 risk stratification failed to identify high‑risk cases; therefore, it is possible that the chemotherapy induction intensity was inadequate. We believe that chemotherapy de‑escalation represents a future trend but may not be universally applicable; intragroup heterogeneity deserves more attention, and early intensification may benefit SR patients within the heterogeneous subgroup harboring risk factors at diagnosis.

Heterogeneity is key to prognostic research on *ETV6*::*RUNX1*‑positive ALL, with proven intrinsic differences in clonal evolution and molecular and pharmacological profiles [4, 13, 14]. Focusing on clinical applicability, affordability and timeliness, we analyzed clinical and immunophenotypic heterogeneity at diagnosis and its correlation with D15/D33-MRD and clinical events to screen potential risk predictors. MRD is a reliable indicator of early treatment response and a cornerstone of risk stratification [10, 15]. In the IR/HR group, D15/D33-MRD status was not correlated with long‑term prognosis, nor was D15-MRD associated with subsequent D33-MRD, suggesting that risk‑adapted therapy offset the negative impact of MRD positivity. In contrast, SR patients who were positive for D15/D33-MRD had significantly higher adverse event rates, underscoring the limitations of the current stratification and the need for earlier risk‑upgrading indicators. Among the immunophenotypic indicators assessed in this study, CD33 positivity at diagnosis was identified as an independent risk factor for D33-MRD positivity, a finding that attracted our attention.

CD33, a sialic acid‑binding immunoglobulin‑like lectin encoded at 19q13.3, is absent on pluripotent stem cells but expressed on early hematopoietic progenitors; it is present in ~ 50% of *ETV6*::*RUNX1*‑positive ALL patients, which is consistent with our observations at diagnosis [5, 16]. While CD33 is a validated prognostic and therapeutic target in acute myeloid leukemia (AML) [17, 18], its role in ALL remains controversial [19–21]. Studies by Pui et al. revealed that myeloid antigen expression is nonprognostic, whereas Cantù‑Rajnoldi et al. linked it to poor outcomes [22–24]. Our findings may offer a reasonable explanation for the above discrepancy: the prognostic value of CD33 is not absolute. In the present study, CD33 expression was not significantly directly correlated with clinical events. However, CD33 expression may serve as a useful auxiliary reference indicator for identifying high-risk patients in the early stage of treatment, especially when combined with D15-MRD-positive status. That is, the CD33⁺/D15‑MRD⁺ subgroup may represent a population that warrants early intensified intervention. A study by Jeha et al. in 2021 demonstrated that D15-MRD can effectively identify patients with poor early treatment response who would otherwise be misclassified into the low-risk group, highlighting the potential clinical value of D15-MRD [25]. Our study revealed that the adverse prognostic impact of D15-MRD positivity was significant only in the CD33-positive subgroup, whereas no clear effect was observed in the CD33-negative subgroup. These findings align with the concept of precision medicine, which holds that combining biological markers with dynamic treatment response indicators enables finer disease risk stratification [15]. Notably, 13 of the 19 patients with clinical events belonged to the CD33⁺/D15‑MRD⁺ subgroup. Among these 13 patients, 10 continued to receive SR chemotherapy after day 15 of treatment. Although most of these patients underwent risk escalation by day 33, clinical events still occurred. The insufficient intensity of induction therapy in this specific subgroup may be an important contributing factor. Previous studies have indicated that insufficient induction chemotherapy intensity is associated with inadequate clearance of leukemic blasts and directly increases the risk of relapse [10, 26, 27]. Therefore, our data suggest that for patients with a CD33⁺/D15‑MRD⁺ status, continuing SR chemotherapy may be suboptimal, and implementing early intensified intervention—such as risk‑escalated therapy or targeted therapy on day 15—could help reduce the occurrence of clinical events.

As a retrospective study, our work has several limitations. The sample size in the IR/HR group was relatively small, and next‑generation sequencing data were unavailable since sequencing was not included as a routine or mandatory test in the CCLG‑ALL 2008 protocol, thus limiting a more comprehensive analysis of genomic characteristics. In addition, the small number of clinical events may have affected the statistical power and robustness; nevertheless, even a marginal trend still merits clinical concern for this molecular subtype.

In conclusion, pediatric *ETV6*::*RUNX1*‑positive ALL patients have an overall favorable prognosis but exhibit intragroup heterogeneity. For patients with this subtype who are CD33‑positive at diagnosis, close monitoring of D15-MRD status is warranted. If positive for D15-MRD, we suggest that continuing SR chemotherapy alone may be insufficient and that early intervention should be considered to reduce the risk of clinical events resulting from insufficient chemotherapy induction intensity. For these patients, targeted therapy may represent a highly promising intervention, as CD33‑targeted therapy has been well‑established and clinically validated for AML, although its applicability for ALL requires dedicated investigation [28, 29]. Although the evidence in the present study is insufficient to support CD33 expression as an independent risk factor for the *ETV6*::*RUNX1*‑positive ALL subtype, its combination with MRD status may serve as a useful auxiliary reference for the early identification of high-risk patients with this subtype. Notably, D15-MRD status has already been incorporated into risk assessment in the updated CCLG‑ALL 2018 protocol, and our findings, if validated in expanded cohorts, could help refine such strategies and further improve the prognosis in patients with this ALL subtype.

## Supplementary Information

Below is the link to the electronic supplementary material.

Supplementary Material 1

## Data Availability

The data that support the findings of this study are available on request from the corresponding authors (by email).

## Abbreviations

ALL: acute lymphoblastic leukemia
AML: acute myeloid leukemia
CCLG: chinese children’s leukemia group
CI: confidence interva
EFS: event free survival
HR: high risk
IR: intermediate risk
IRB: institutional review board
ROC: receiver operating characteristic
MICM: morphology, immunology, cytogenetics and molecular biology
MRD: minimal residual disease
OS: overall survival
OR: odds ratiol
SR: standard risk
