# Supplementary material for: CD33 expression combined with D15-MRD positivity identifies poor prognosis in children with ETV6::RUNX1-positive ALL
Source: Ann Hematol. 2026 May 29;105(8):347. doi: 10.1007/s00277-026-07089-8 (PMC13408124; doi:10.1007/s00277-026-07089-8)
Supplement: Supplementary file 1 — Supplementary Material 1 [file 277_2026_7089_MOESM1_ESM.docx]

**Supplementary Data1**

**Experimental details of quantitative real-time polymerase chain reaction (qRT-PCR) for molecular MRD detection:**

For sample processing, total RNA was extracted from bone marrow mononuclear cells (EDTA-anticoagulated) using TRIzol reagent, followed by reverse transcription into cDNA (final concentration, 80 ng/μL).

The *ETV6::RUNX1* primer–probe set was as follows:

Forward: CTCTGTCTCCCCGCCTGAA;

Reverse: CGGCTCGTGCTGGCAT; and

Probe (FAM-TAMRA labeled): TCCCAATGGGCATGGCGTGC.

The internal control was ABL/GUS, a housekeeping genes recommended by EuroMRD for copy number normalization.

The PCR protocol was as follows: 95 °C for 15 min followed by 50 cycles of 95 °C for 15 s and 60 °C for 60 s.For quality control, triplicate wells for target genes and duplicate wells for the internal control were used.

**Supplementary Data2**

The results of logistic regression for multivariate analysis

|  | OR（95%CI） | P |
| --- | --- | --- |
| MRD-D15 (all patients) | | |
| CD33+ | 2.798（1.734-4.514） | ＜0.001 |
| CD4+ Th |  | 0.016 |
| Low | 2.651（1.362-5.162） | 0.004 |
| High | 0.976（0.153-6.221） | 0.979 |
| WBC | 1.006（0.997-1.014） | 0.180 |
| Adolescent | 0.915（0.197-4.240） | 0.910 |
| MRD-D33 (SR) | | |
|  |  |  |
| CD33+ | 1.974（1.122-3.471） | 0.018 |
| CD22+ | 0.464（0.266-0.810） | 0.007 |
| WBC | 1.027（0.995-1.061） | 0.102 |
| MRD-D15 | 4.425（2.468-7.934） | ＜0.001 |
| Adolescent | - | - |
| MRD-D33 (IR+HR) | | |
|  |  |  |
| CD33+ | 1.931（0.611-6.096） | 0.262 |
| CD22+ | 0.272（0.080-0.932） | 0.038 |
| WBC | 0.998（0.989-1.008） | 0.726 |
| MRD-D15 | 0.867（0.255-2.950） | 0.819 |
| Adolescent | 5.970（0.543-65.679） | 0.144 |

**Supplementary Data3**

The variable importance of predictors in the decision tree stratified by the CD33

|  | predictors | importance of predictors(%) |
| --- | --- | --- |
| CD33 positive | MRD_15 | 33 |
|  | Bone destruction | 17 |
|  | LN | 13 |
|  | CD22 | 9 |
|  | CD20 | 8 |
|  | MRD_33 | 7 |
|  | CD_17 | 2 |
|  | CyIgM | 2 |
|  | CD34 | 1 |
|  | Hepatomegaly | 1 |
| CD33 negative | MRD_33 | 33 |
|  | Hepatomegaly | 17 |
|  | LN | 14 |
|  | CD20 | 13 |
|  | CD22 | 6 |
|  | CD117 | 5 |
|  | Splenomegaly | 5 |
|  | MRD_15 | 4 |
|  | CD34 | 2 |
|  | CD56 | 1 |


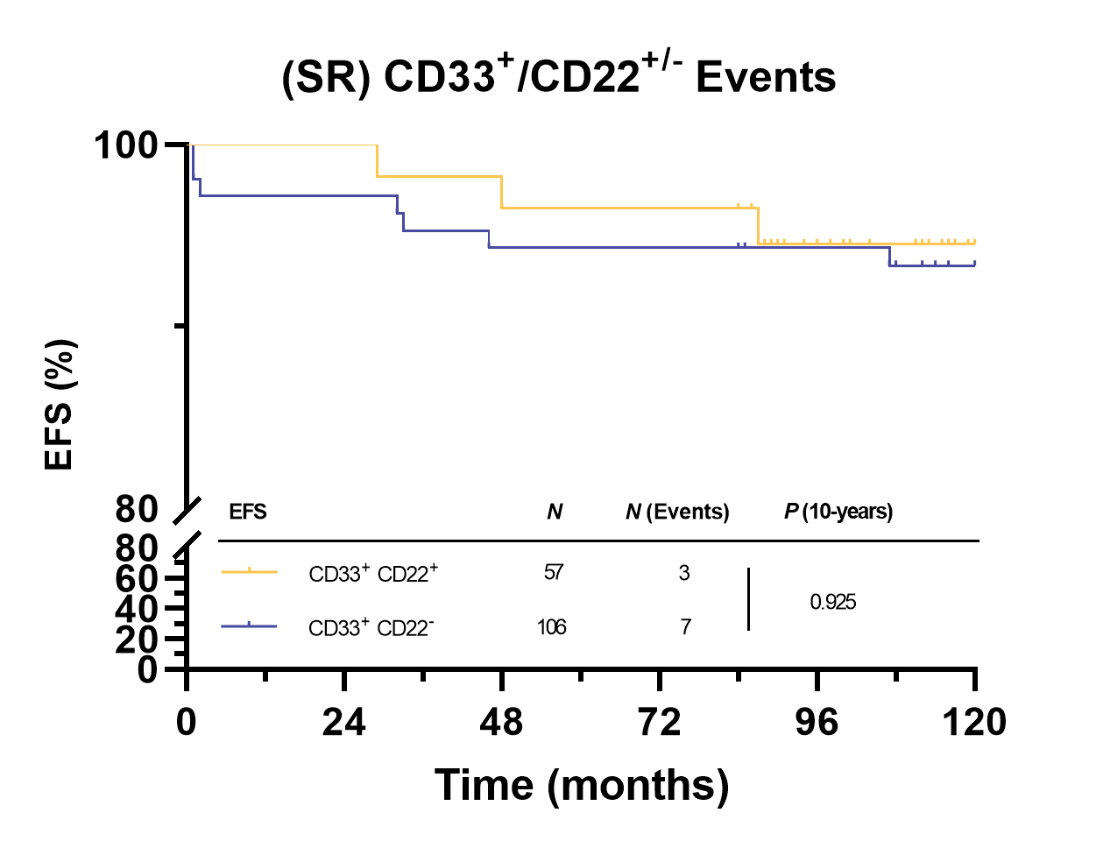


Supplementary Data4 EFS of *ETV6*::*RUNX1*-positive ALL patients according to CD33^+^/CD22 ^+/-^ status in the standard-risk group.
